# Effects of Benzo[*a*]pyrene Exposure on Human Hepatocellular Carcinoma Cell Angiogenesis, Metastasis, and NF-**κ**B Signaling

**DOI:** 10.1289/ehp.1408524

**Published:** 2014-10-17

**Authors:** Qian Ba, Junyang Li, Chao Huang, Hongling Qiu, Jingquan Li, Ruiai Chu, Wei Zhang, Dong Xie, Yongning Wu, Hui Wang

**Affiliations:** 1 Key Laboratory of Food Safety Research, Institute for Nutritional Sciences, Shanghai Institutes for Biological Sciences, Chinese Academy of Sciences, Shanghai, China; 2 Key Laboratory of Food Safety Risk Assessment, Ministry of Health, Beijing, China; 3 Institute for Food Safety and Health, Illinois Institute of Technology, Bedford Park, Illinois, USA; 4 School of Life Science and Technology, ShanghaiTech University, Shanghai, China

## Abstract

**Background:**

Benzo[*a*]pyrene (B[a]P) is a common environmental and foodborne pollutant. Although the carcinogenicity of high-dose B[a]P has been extensively reported, the effects of long-term B[a]P exposure at lower environmental doses on cancer development are less understood.

**Objectives:**

We investigated the impact of B[a]P on human hepatocellular carcinoma (HCC) progression at various levels of exposure and identified a potential intervention target.

**Methods:**

We used a model based on human HCC cells exposed to various concentrations of B[a]P (i.e., 0.01, 1, or 100 nM) for 1 month to examine the effects of B[a]P on cell growth, migration, invasion, and angiogenicity. A bioluminescent murine model was established to assess tumor metastasis *in vivo*.

**Results:**

Chronic B[a]P exposure did not alter HCC cell growth but promoted cell migration and invasion both *in vitro* and *in vivo*. There was an negative association between B[a]P exposure and the survival of tumor-bearing mice. In addition, B[a]P-treated HCC cells recruited vascular endothelial cells and promoted tumor angiogenesis, possibly through elevating vascular endothelial growth factor secretion. Furthermore, the NF-κB pathway may be an adverse outcome pathway associated with the cumulative effects of B[a]P on HCC metastasis.

**Conclusions:**

These findings *a*) indicate that B[a]P has effects on HCC progression; *b*) identify a possible adverse outcome pathway; and *c*) contribute to a better understanding of the adverse effects of chronic exposure of B[a]P to human health.

**Citation:**

Ba Q, Li J, Huang C, Qiu H, Li J, Chu R, Zhang W, Xie D, Wu Y, Wang H. 2015. Effects of benzo[*a*]pyrene exposure on human hepatocellular carcinoma cell angiogenesis, metastasis, and NF-κB signaling. Environ Health Perspect 123:246–254; http://dx.doi.org/10.1289/ehp.1408524

## Introduction

Benzo[*a*]pyrene (B[a]P), a prototypical and well characterized member of the polycyclic aromatic hydrocarbon (PAH) family (Phillips 1983; Srivastava et al. 2000), is a procarcinogen formed in the process of incomplete combustion of organic materials (Gelboin 1980). Human contact with B[a]P from the environment is unavoidable (Phillips 1999; Srogi 2007). As a ubiquitous environmental and foodborne pollutant, B[a]P is found widely in engine exhaust, tobacco smoke, charcoal-grilled foods, and contaminated water and soil (Phillips 1999). B[a]P enters the human body mainly through inhalation and ingestion and is transported to other organs by blood and lymph (Van de Wiele et al. 2005). Once taken up into cells, B[a]P undergoes metabolic activation by the cytochrome P450-dependent monooxygenase system and is converted to reactive, toxic metabolites that bind covalently to cellular elements such as DNA (Rivedal and Sanner 1981; Rubin 2001). B[a]P metabolism also generates reactive oxygen species, which damage cellular macromolecules (Rubin 2001; Umannová et al. 2011).

The adverse effects of B[a]P, including carcinogenicity, teratogenicity, neurotoxicity, and immunotoxicity, on various species of experimental animals have been described previously (Davila et al. 1996; Mendola et al. 2002; Min et al. 2011; Wolterbeek et al. 1995). B[a]P, which induces tumors in multiple organs of laboratory animals, is categorized as a human Group 1 carcinogen by the International Agency for Research on Cancer (IARC) (Einem Lindeman et al. 2011; IARC 2010). The location of tumors appears to be related to the route of exposure. Inhalation of B[a]P often induces lung cancer, and oral administration leads to tumors in various organs/tissues, including the gastrointestinal tract, liver, lungs, and mammary glands (Benford et al. 2010). In epidemiological studies, PAH–albumin and PAH–DNA adducts, which reflect PAH exposure, are associated with an elevated risk of hepatocellular carcinoma (HCC) (Chen et al. 2002; Wu et al. 2007). Moreover, environmental exposure to high levels of B[a]P increases the risk of HCC, suggesting that B[a]P might be a cause of HCC (Su et al. 2014).

However, most information on B[a]P toxicity has been obtained from animal studies, and the extrapolation from laboratory animals to humans is uncertain. In addition to species differences, extrapolating the toxicological effects of high B[a]P doses in animals to effects that might occur at environmentally relevant concentrations in humans is problematic. For this reason, the traditional strategy of using high-dose B[a]P to evaluate toxicity is not conclusive for an understanding of the carcinogenic mechanism of B[a]P in humans. The U.S. National Research Council proposed Toxicity Testing in the 21st Century (TT21C), which encouraged a transformation of toxicity testing from high-dose studies in laboratory animals to *in vitro* toxicity pathway–based approaches using human-relevant cells (Adeleye et al. 2014; Bhattacharya et al. 2011; Gibb 2008). These *in vitro* assays can be used to evaluate the responses of toxicity pathways [or adverse outcome pathways (AOP)], which are innate cellular signaling pathways, and would result in adverse cellular outcomes if perturbed (Adeleye et al. 2014; Bhattacharya et al. 2011; Gibb 2008). However, how to apply the AOP/TT21C strategy in B[a]P toxicity testing is still under investigation.

Some populations, such as disease groups, may be more susceptible to B[a]P exposure than healthy groups. Thus, the cumulative adverse health effects of lower-dose B[a]P on susceptible populations should be considered and investigated. Although numerous studies have illustrated the effects of B[a]P on malignant transformation and carcinogenesis (Benford et al. 2010; Su et al. 2014; Wolterbeek et al. 1995), the potential roles of B[a]P, especially low-dose B[a]P exposure, on cancer aggressiveness and progression are rarely reported.

In the present study, we examined the chronic toxicity of B[a]P using human-derived HCC cell lines that were subjected to long-term B[a]P exposure at environmental-relevant concentrations. We determined the biological effects of B[a]P on cancer metastasis and progression, explored the adverse outcome pathway, and identified the NF-κB pathway as a potential target.

## Materials and Methods

*Cell cultures and regents*. Human HCC cell lines, SMMC-7721 and BEL-7404, and human umbilical vein endothelial cells (HUVECs) were obtained from the Cell Bank of the Shanghai Institutes for Biological Sciences, Chinese Academy of Sciences (SIBS CAS) and cultured in RPMI 1640 medium supplemented with 10% fetal bovine serum, 100 μg/mL penicillin, and 100 μg/mL streptomycin and maintained in an incubator with a humidified atmosphere of 5% CO_2_ at 37°C. For B[a]P exposure, BEL-7404 and SMMC-7721 cells were co-cultured with 0.01 nM, 1 nM, 100 nM B[a]P, or 0.1% DMSO for up to 1 month. After treatment, B[a]P was withdrawn and the effects of B[a]P on HCC progression were determined. Cell morphology was observed using an inverted microscope. We used the Cell Counting Kit-8 (CCK-8) (Dojindo, Shanghai, China) to measure cell growth. BAY11-7085 (an NF-κB inhibitor) was purchased from Gene Operation, Inc. (Ann Arbor, MI, USA). B[a]P, propidium iodide, crystal violet, and other chemicals used in this study were purchased from Sigma-Aldrich Inc. (St. Louis, MO, USA).

*Western blot assays*. Western blot analyses were conducted as described previously (Ba et al. 2012). Total cellular proteins were separated and probed with specific antibodies. We purchased E-cadherin antibody from BD Biosciences (San Jose, CA, USA) and N-cadherin antibody from Upstate (Billerica, MA, USA). Anti-human vimentin, snail, slug, and β-actin antibodies were obtained from Sigma-Aldrich. Antibodies against phosphorylated NF-κB p65 and total NF-κB p65 were purchased from Cell Signaling Technology Inc. (Danvers, MA, USA). All other antibodies were purchased from Santa Cruz Biotechnology (Santa Cruz, CA, USA). Nuclear and cytoplasmic protein extractions were performed using a commercial kit (Thermo Fisher Scientific, Waltham, MA, USA).

*Cell cycle analysis*. Cell cycle distributions were analyzed as described previously (Ba et al. 2011). BEL-7404 cells were harvested and suspended in 70% cold ethanol, then incubated overnight at 4°C. After centrifugation, the pellets were washed with cold phosphate-buffered saline (PBS), suspended in 500 μL PBS, and incubated with 50 μL RNase A (20 μg/mL final concentration) for 30 min. The cells were incubated with propidium iodide (50 μg/mL) for 30 min in the dark. Cell cycles were determined using a FACSAria instrument (BD Biosciences) (Ba et al. 2011).

*Soft agar assay*. For determination of anchorage-independent growth, BEL-7404 cells were plated into 6-well plates containing two layers of soft agar. The base layer was prepared with 0.5% agar. Approximately 2,500 cells were suspended in medium with 0.35% agar and seeded over the base layer. Colony formation was monitored daily by microscopic observation. After incubation at 37°C for 2 weeks, the size of colonies in 6-well plates was photographed directly with an inverted microscope.

*Cell migration and invasion assays*. For the migration assay, BEL-7404 and SMMC-7721 cells in serum-free medium were seeded in Transwell Permeable Supports with 8-μm microporous membranes (Corning, NY, USA) in 24-well plates. The lower compartments of the plates were filled with medium containing 10% fetal bovine serum. After incubation for 18 hr, cells on the upper surface of the membrane were removed; cells that migrated to the lower surface and across the filters were fixed and stained with eosin or crystal violet in methanol, and counted after photography under a microscope. For the invasion assay, Transwell inserts were precoated with Matrigel (1.25 mg/mL; BD Biosciences). BEL-7404 and SMMC-7721 cells in serum-free medium were seeded on the insert and incubated for 48 hr. Cells that invaded to the lower surface of membrane were stained and counted as described above.

*Cell adhesion assay*. BEL-7404 and SMMC-7721 cells were plated into 96-well plates precoated with Matrigel. After incubation for 30 min at 37°C, the medium was discarded and the cells were washed twice with PBS to remove the nonadherent cells. The attached cells were incubated with CCK-8 in medium for 4 hr and quantified by measuring the absorbance at 450 nm with a SpectraMax 190 microplate reader (Molecular Devices, Sunnyvale, CA, USA).

*Animal husbandry*. All animals were treated humanely and with regard for alleviation of suffering according to the Biomedical Research Ethics Committee of the SIBS, CAS.

Twelve male BALB/c nude mice (4 weeks of age) were obtained from the Shanghai Laboratory Animal Research Center (Shanghai, China) and housed in the mouse barrier facility of the Institute for Nutritional Sciences, SIBS CAS. Animals were housed three per cage by group on corncob bedding in an individually ventilated caging system under a 12:12 hr light:dark cycle, with a humidity of 40–70% and the room temperature at 20–24°C. All mice were fed with a standard laboratory rodent diet irradiated with cobalt-60 (SLRC Laboratory Animal Co. Ltd., Shanghai, China) and reverse osmosis water *ad libitum*. In the experiment, the mice were subjected to tail vein injection with SMMC-7721 cells (1 × 10^6^) and intraperitoneal injection with D-luciferin (300 mg/kg body weight), and they were anesthetized with isoflurane gas. All procedures were conducted in the afternoon in each animal’s home cage.

In vivo *metastasis assay*. SMMC-7721 cells exposed to various concentrations of B[a]P for 1 month were labeled with luciferase-expressing lenti-virus containing an independent open-reading frame of green fluorescent protein (GFP). Infection-positive cells were collected through cell sorting by flow cytometry. Luciferase expression was determined using D-luciferin and *in vivo* imaging (IVIS Lumina Imaging System; Xenogen, Baltimore, MD, USA). The luciferase-expressing SMMC-7721 cells (1 × 10^6^ in serum-free medium) were delivered to nude mice by tail vein. Luciferase activity was monitored weekly by intraperitoneal injection of D-luciferin (300 mg/kg body weight). At 30 min after injection, animals anesthetized with isoflurane were placed in a dark imaging chamber and imaged. The results were analyzed with an IVIS Lumina Imaging System. Photons from the luciferin/luciferase reaction were collected with a CCD camera. Photon signals of equal size were quantified using Living Image® software (Xenogen).

*Angiogenesis assays*. We performed angiogenesis assays using the tube formation method (Arnaoutova and Kleinman 2010). Briefly, 96-well plates were precoated with 80 μL/well growth factor-reduced Matrigel. HUVECs were washed with PBS and seeded at 1.5 × 10^4^ cells/well in the presence of conditioned media. After incubation for 6 hr, images of capillary-like structures were captured with an inverted microscope. Relative quantities of the tubules were quantified by Angiogenesis Analyzer for Image J software (http://imagej.nih.gov/ij/) (Gilles 2012).

*S9 mixture reaction and enzyme-linked immunosorbent assay (ELISA)*. BEL-7404 and SMMC-7721 cells were harvested in PBS. After sonication, the S9 fraction was obtained by centrifuging at 9,000 rcf (relative centrifugal force) for 20 min. An inactivated S9 fraction was prepared by boiling for 15 min. The S9 reaction system containing NADPH (5 mM), bovine serum albumin (BSA; 0.4 mg/mL), B[a]P (10 μg/mL), and S9 fraction was incubated for 2 hr at 37°C. To test whether B[a]P could be activated by the S9 fraction, the concentrations of benzo[*a*]pyrene diol epoxide (BPDE) were determined using Human BPDE ELISA kits (AMEKO; Shanghai Lianshuo Biological Technology Co. Ltd., Shanghai, China) according to the manufacturer’s instructions. The concentration of vascular endothelial growth factor (VEGF), the main proangiogenic factor, were measured in culture media by Human VEGF ELISA kits (MR Biotech, Shanghai, China) according to the manufacturer’s instructions. Absorbance at 450 nm was recorded with a SpectraMax 190 microplate reader.

*Immunofluorescence assay*. SMMC-7721 cells were seeded on glass coverslips. After attachment, cells were fixed with 4% paraformaldehyde in PBS for 10 min, and permeabilized with 0.1% Triton X-100 in PBS for 10 min. After washing with PBS, the coverslips were blocked with 3% BSA and incubated with primary antibody against NF-κB p65 and then with the secondary antibody Alexa Fluor 555 donkey anti-rabbit IgG (Molecular Probes, Grand Island, NY, USA). After three washes, the coverslips were mounted with ProLong Gold antifade reagent (Invitrogen, Carlsbad, CA, USA) and sealed with nail polish. Images of p65 cellular distribution were captured with a fluorescence microscope (Olympus, Tokyo, Japan).

*Luciferase reporter assay*. BEL-7404 cells were seeded at a subconfluent density and cotransfected with the NF-κB reporter construct and the Renilla luciferase plasmid, which was the internal control for transfection efficiency. Cells were lysed, and NF-κB reporter activity was measured with the Dual-Luciferase Reporter Assay System (Promega, Madison, WI, USA) according to the manufacturer’s protocol.

*Statistical analyses*. Data are presented as the mean ± SD. The statistical significance of differences was examined using Student’s *t*-test. We used one-way analysis of variance (ANOVA) and test for linear trend to analyze the dose–response relationship (GraphPad Prism, version 6.01; GraphPad Software Inc.; La Jolla, CA, USA). Data from *in vivo* metastasis assays were analyzed by two-way ANOVA, and Tukey’s multiple comparisons test was used to analyze the difference between groups. Survival curves were established using Kaplan-Meier methodology and analyzed using the log-rank test for trend. *p* < 0.05 was considered statistically significant.

## Results

*HCC cell growth*. To investigate the potential chronic toxicity of B[a]P, low-dose and long-term exposure models were established with the human HCC cell lines BEL-7404 and SMMC-7721, which retained the capacity to metabolically activate B[a]P (see Supplemental Material, Figure S1).

**Figure 1:**
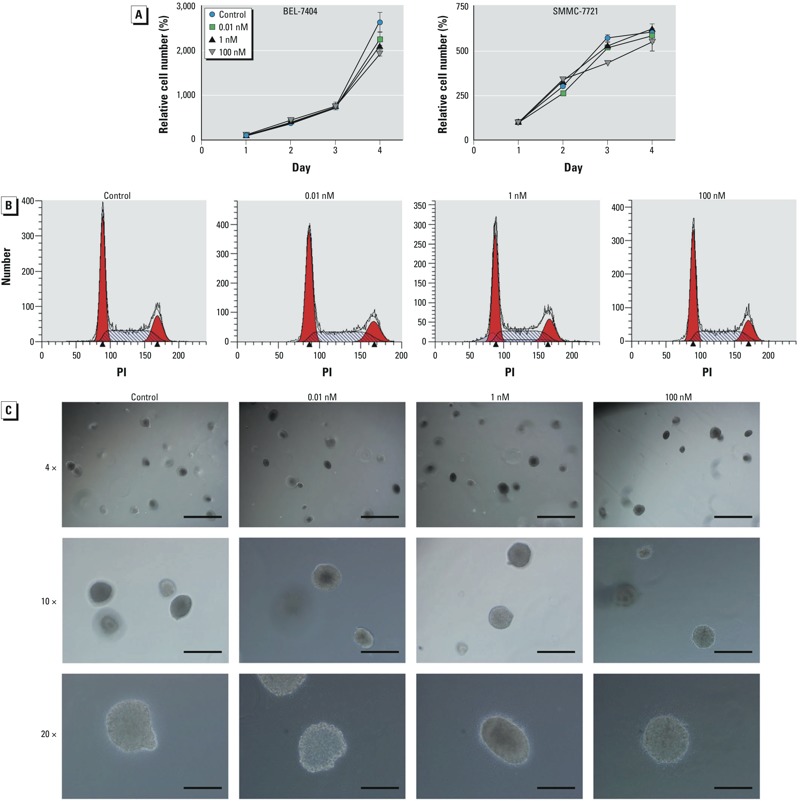
Long-term exposure to B[a]P showed no detectable effects on HCC cell growth. (*A*) BEL-7404 (left) and SMMC-7721 (right) cells were treated with 0.1% DMSO or different concentrations of B[a]P for 1 month, and cell growth was evaluated by the CCK-8 assay; values are mean ± SD (*n *= 6/group). (*B*) BEL-7404 cells were treated with 0.1% DMSO or B[a]P for 1 month and harvested for cell cycle distribution analysis by flow cytometry. Red areas represent G_1_/S and G_2_/M phases; arrowheads indicate the peaks of G_1_/S and G_2_/M phases. (*C*) Soft agar assay of BEL-7404 cells exposed to 0.1% DMSO or different concentrations of B[a]P for 1 month. Bars = 1 mm for 4× magnification (top), 400 μm for 10× magnification (center), and 200 μm for 20× magnification (bottom).

The doses included a high concentration for testing multiple modes of action, and the low concentration was at an environmental exposure level. In the B[a]P exposure models, BEL-7404 and SMMC-7721 cells were cocultured with 0.01, 1 nM, and 100 nM B[a]P or 0.1% DMSO for up to 1 month. In studies involving cultures, no evident morphological abnormalities were observed (see Supplemental Material, Figure S2), and all groups of cells exhibited comparable growth rates. To verify this, cell proliferation assays were performed for BEL-7404 and SMMC-7721 cells. The growth curves showed similar patterns between control and B[a]P-treated groups (Figure 1A). B[a]P exposure did not change the cell cycle distributions of BEL-7404 cells (Figure 1B). Further, the anchorage-independent growth of BEL-7404 cells was determined by a soft agar assay. All groups formed cell clones in soft agar, and no variation in clone formation was observed (Figure 1C). These results indicate that in human HCC cells, sustained exposure of B[a]P has no detectable effect on anchorage-dependent or -independent cell growth.

**Figure 2:**
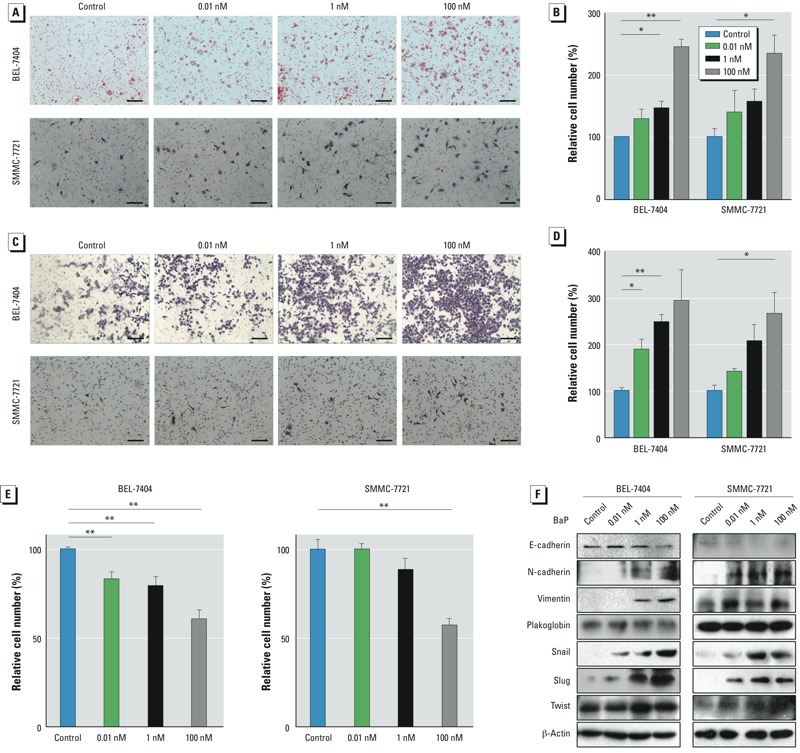
B[a]P-exposed HCC cells showed high metastatic proclivity. (*A*) Representative images from cell migration assays; bars = 150 μm. (*B*) The numbers of migrated cells were calculated and normalized to the control group; values are mean ± SD (*n* = 3/group; *p* for trend = 0.0003 for BEL‑7404 cells and 0.0079 for SMMC‑7721 cells). (*C*) Representative images of cell invasion identified using the Matrigel invasion assay; bars = 150 μm. (*D*) Numbers of invasive cells calculated and normalized to the control group; values are mean ± SD (*n* = 3/group; *p* for trend = 0.0057 for BEL‑7404 cells and 0.0043 for SMMC‑7721 cells). (*E*) Cells were plated into 96-well plates precoated with Matrigel and incubated for 30 min at 37°C, and cell adhesion levels were determined and normalized to the control group. Values are mean ± SD (*n* = 6/group; *p* for trend = 0.0001 for both BEL‑7404 and SMMC‑7721 cells). (*F*) Western blots of total cellular proteins. *p < 0.05, and **p < 0.01.

*HCC cell migration and invasion*. To investigate the effects of long-term B[a]P exposure on cancer progression, we evaluated the mobility of B[a]P-treated HCC cells. After sustained B[a]P exposure for 1 month, the migration of both BEL-7404 and SMMC-7721 cells were significantly increased in a dose-dependent manner (Figure 2A,B). In addition, cell invasion was enhanced by B[a]P treatment, even at a low concentration (Figure 2C,D). The adhesion of B[a]P-treated BEL-7404 and SMMC-7721 cells was determined using Matrigel to induce adhesion. The numbers of adherent cells were reduced with increasing concentrations of B[a]P (Figure 2E). These results indicate that B[a]P suppressed HCC cell adhesion to an extracellular matrix, which may partially explain the increased cell mobility induced by B[a]P. Moreover, prolonged treatment of B[a]P for 1 month altered the expression of cancer metastasis-related proteins in HCC cells. E-cadherin, which inhibits cancer cell migration and invasion, was reduced in B[a]P-treated groups relative to the control group, but expression of metastasis-promoting proteins, including N-cadherin, vimentin, snail, and slug, were induced after B[a]P exposure (Figure 2F).

**Figure 3:**
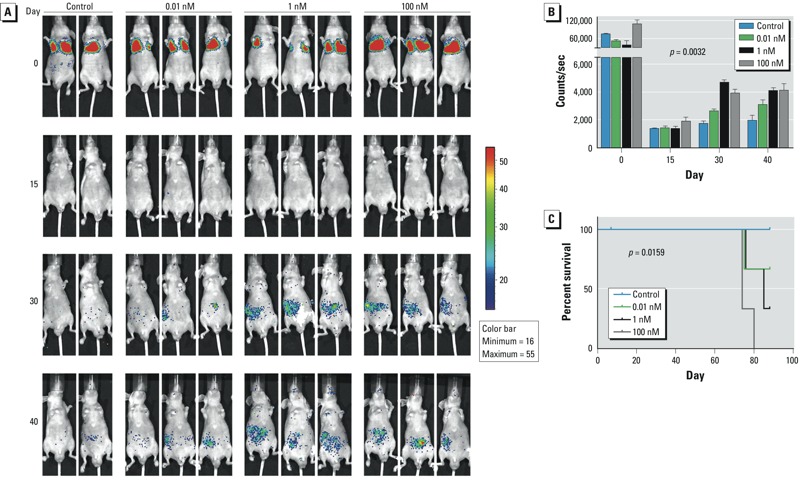
B[a]P-exposed HCC cells metastasized more extensively in nude mice than did control cells. (*A*) Monitoring of metastasis of bioluminescent SMMC-7721 cells exposed to B[a]P; images were obtained at 30 min (0 days), 15 days, 30 days, and 40 days after injection with cells. (*B*) Quantification of photon counts for each group of mice over time; values are mean ± SD (*n* = 2–3/group; *p* = 0.0032 by two-way ANOVA). (*C*) Kaplan-Meier overall survival curves for mice injected with HCC cells exposed to different doses of B[a]P; B[a]P treatment reduced the overall survival rate in a concentration-dependent manner (*p* = 0.0159, log-rank test for trend).

*HCC metastasis* in vivo. To explore the metastatic activity of prolonged doses of B[a]P to HCC cells *in vivo*, we used a bioluminescent murine model. After 1 month of treatment with B[a]P, SMMC-7721 cells were labeled with luciferase by lentivirus infection. After infection, each cell group showed similar luciferase activity (see Supplemental Material, Figure S3A). No impact of virus infection on the migration-promoting effects of B[a]P was observed (see Supplemental Material, Figure S3B). After luciferase labeling, cells exposed to different concentrations of B[a]P were injected into the tail veins of nude mice, which were randomly divided into four groups. The subsequent metastases of HCC cells were measured weekly by luciferin intensity. Consistent with the results for HCC cell lines, B[a]P-treated groups showed more metastatic cancer cells in a concentration-dependent manner (*p* = 0.0032) (Figure 3A,B), indicating that 1 month of exposure to B[a]P could enhance HCC metastasis. Moreover, the survival of tumor-bearing mice was associated with B[a]P exposure and concentration (*p* = 0.0159). With increasing B[a]P concentrations, the survival of mice declined significantly (Figure 3C). These findings suggest that sustained exposure of B[a]P, even at a low dose, promotes HCC progression both *in vitro* and in mice.

**Figure 4:**
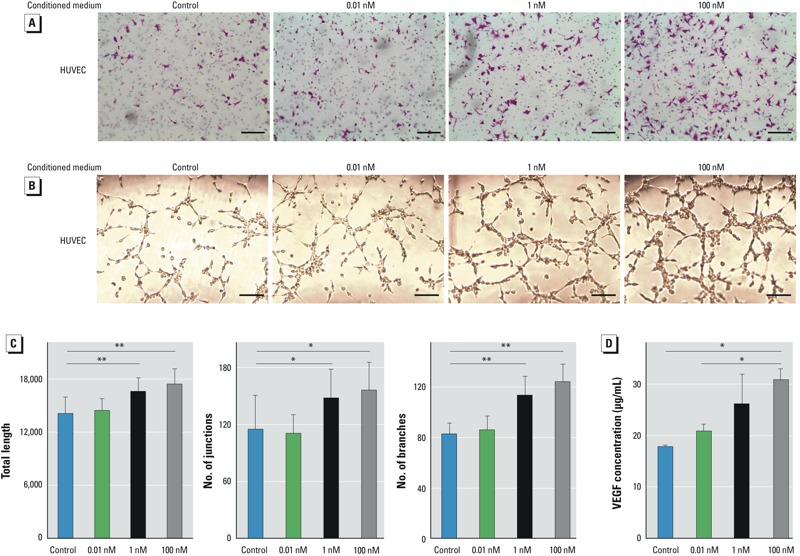
Long-term exposure of B[a]P-enhanced tumor-associated angiogenesis *in vitro*. (*A*) Representative images showing the effect of conditioned medium from B[a]P-exposed BEL-7404 cells on HUVEC recruitment. Transwell plates containing HUVECs in the top chambers and conditioned medium from B[a]P-exposed BEL-7404 cells in the bottom chambers were incubated for 6 hr; bars = 150 μm. (*B*) Representative images showing the effect of conditioned medium from B[a]P-exposed BEL-7404 cells on tube formation. HUVECs were seeded on top of Matrigel with reduced growth factors and incubated for 6 hr in conditioned media. bars = 200 μm. (*C*) Quantitation of the angiogenesis assay by ImageJ softwarel; values are mean ± SD (*n* = 3/group; *p* for trend = 0.0081 for total length, 0.0282 for number of junctions, and 0.0002 for number of branches). (*D*) Concentrations of VEGF protein in the conditioned media; values are mean ± SD (*n* = 3/group; *p* for trend = 0.0127). *p < 0.05, and **p < 0.01, by Student’s *t*-test.

*Vascular endothelial cell recruitment and angiogenesis*. Because angiogenesis is essential for tumor progression and metastasis, we used HUVECs to examine the influence of chronic B[a]P exposure on tumor angiogenesis. We used supernatants of culture media from BEL-7404 cells exposed to different concentrations of B[a]P. The induction effects of conditioned media on HUVECs were determined by Transwell assays. Relative to control medium derived from BEL-7404 cells, the conditioned media from B[a]P-exposed BEL-7404 cells attracted more HUVECs in a dose-dependent manner (Figure 4A). Furthermore, the conditioned media from B[a]P-exposed BEL-7404 cells markedly induced more tube formation (Figure 4B). The junctions and branches, and the total length of tubes were increased significantly in B[a]P-treated groups (Figure 4C). To further investigate the angiogenesis-promoting effect of B[a]P, we used ELISA to measure the concentration of VEGF, the main proangiogenic factor, in conditioned media. Consistent with the above results, 1 month exposure of B[a]P promoted VEGF secretion by BEL-7404 cells in a concentration-dependent manner (Figure 4D). Thus, B[a]P-exposed HCC cells were more capable of recruiting vascular endothelial cells and enhancing angiogenesis.

**Figure 5:**
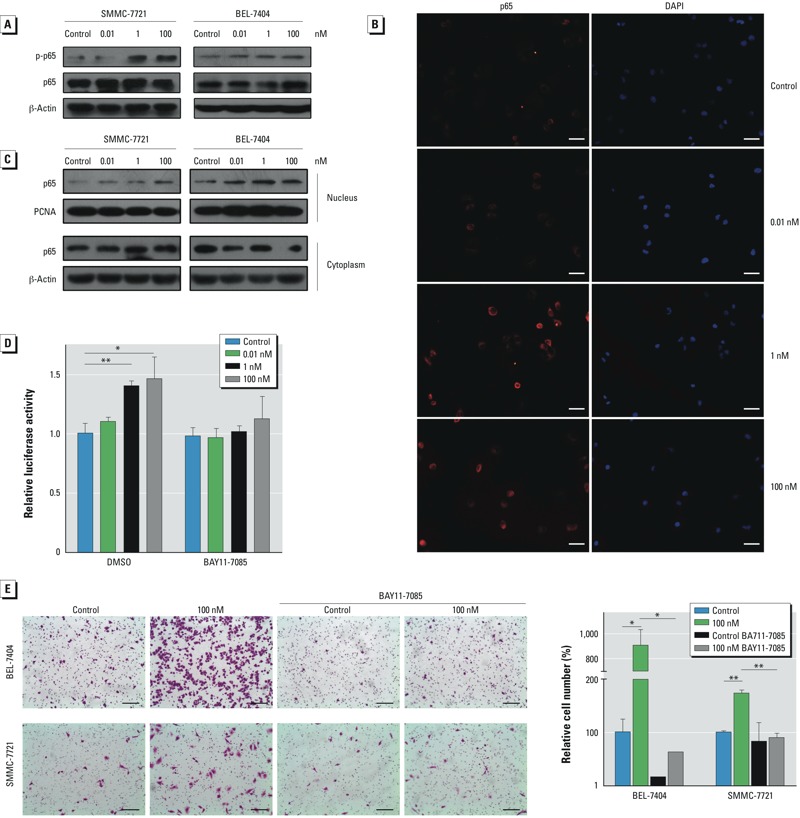
B[a]P promoted HCC cell metastasis through activating the NF-κB signaling pathway. (*A*) Western blots showing phosphorylated p65 (p‑p65), p65, and β‑actin (loading control) in B[a]P-treated BEL-7404 and SMMC-7721 cells. (*B*) Subcellular location of p65 in B[a]P-treated SMMC-7721 cells was determined by immunofluorescent staining; bars = 100 μm. (*C*) Western blots showing nuclear and cytoplasmic p65; β-actin and proliferating cell nuclear antigen (PCNA) served as loading controls. (*D*) NF-κB promoter activity measured by luciferase assays in B[a]P-treated BEL-7404 cells that were pretreated with BAY11-7085 (5 μM) or DMSO for 6 hr; values are mean ± SD (*n* = 3/group; *p* for trend = 0.0004 for DMSO and 0.1275 for BAY11-7085). (*E*) Cell migration in the presence or absence of BAY11-7085 (5 μM) examined by Transwell assays (left; bars = 150 μm) and the relative numbers of migrated cells (right; values are mean ± SD; *n* = 3/group). *p < 0.05, and **p < 0.01, by Student’s *t*-test.

*NF-*κ*B signaling*. To explore the adverse outcome pathways of B[a]P, we examined several typical signaling pathways and found the NF-κB pathway to be activated during chronic exposure to B[a]P. In both BEL-7404 and SMMC-7721 cells, the expression of phosphorylated p65 (active form) increased in a concentration-dependent manner after B[a]P treatment (Figure 5A). The intracellular distribution of p65 was also altered: In B[a]P-exposed cells, p65 was more likely to translocate into the nucleus (Figure 5B), and the nuclear p65 levels increased markedly (Figure 5C), indicating active regulation of gene expression. Moreover, the promoter activity of NF-κB was elevated by B[a]P treatment in a concentration-dependent manner (Figure 5D), suggesting that long-term exposure of B[a]P resulted in activation of NF-κB signaling. To investigate the role of the NF-κB signaling pathway in B[a]P-induced HCC metastasis, a specific inhibitor of IκBα, BAY11-7085, was utilized to block NF-κB signaling (Figure 5D). After incubation with BAY11-7085, the promoting effects of B[a]P on cell migration were reduced in both BEL-7404 and SMMC-7721 cells (Figure 5E). These results demonstrate an important role of NF-κB signaling pathway in cancer metastasis induced by a month exposure of B[a]P.

## Discussion

In this study, we investigated the potential toxicity of B[a]P, an environmental and foodborne pollutant, on human HCC cells after low-dose and long-term exposure. Our results showed that B[a]P had no effect on HCC cell morphology or growth; however, B[a]P treatment significantly promoted cell migration and invasion, enhanced recruitment of vascular endothelial cells and tumor angiogenesis, activated NF-κB signaling, and elevated VEGF secretion. Overall, low-dose and long-term exposure of B[a]P promoted HCC metastasis and progression *in vitro* and in mouse models. Thus, there were adverse effects of long-term B[a]P exposure on human HCC cells.

To characterize the toxicity of B[a]P, which is difficult to achieve in conventional animal studies, we established a model of the exposure. First, human HCC cells were chosen to avoid extrapolating animal results to humans; the metastatic potential of B[a]P-exposed cells was validated *in vivo* using a mouse imaging system. Second, continuous exposure for 1 month was used to assess cumulative toxicological effects. Third, we used a range of concentrations comparable to the serum B[a]P levels of populations exposed environmentally (≤ 3.88 ± 2.22 nM) (Neal et al. 2008), although how these serum levels would translate to actual tissue levels needs to be investigated. Therefore, our findings provide a better understanding of the toxicity of environmental B[a]P.

As a Group 1 carcinogen listed by the IARC (2010), B[a]P increases the risk of several types of cancers, including those of the lung, gastrointestinal tract, liver, and bladder, in laboratory animals (Benford et al. 2010). Epidemiological findings support an association between the exposure of B[a]P or PAHs and the incidence of lung cancer, colon cancer, and skin cancer (Friesen et al. 2009; Gunter et al. 2007; Tang et al. 1995). B[a]P does not cause cancers until it is metabolized to toxic metabolites by cytochrome P450 enzymes (Rivedal and Sanner 1981; Rubin 2001). Liver tissue has the highest capacity for such biotransformation, making it sensitive to B[a]P exposure. B[a]P administration to experimental animals increases the risk of HCC (Kitagawa et al. 1980; Wills et al. 2010). However, the impact of prolonged B[a]P exposure on HCC development and progression remains unclear. In the present study, we have assessed the effects of B[a]P from the perspective of metastasis and tumor angiogenesis.

Metastasis, the final step of neoplastic progression, remains the major cause of death from HCC (Wang et al. 2008; Winnard et al. 2008). Still, the environmental risk factors for HCC metastasis are not clearly known (Uka et al. 2007). We found that long-term exposure of HCC cells to B[a]P led to more metastatic potential. Both migration and invasion were induced after prolonged B[a]P exposure. Consistently, cell adhesion, a factor related to mobility, was reduced in B[a]P-exposed cells. Evidence indicates that expression of snail, slug, vimentin, and N-cadherin were induced, whereas the level of E-cadherin was inhibited, suggesting that B[a]P might promote the epithelial–mesenchymal transition (EMT) process. The results in mice—consistent with the findings from cell cultures—revealed an effect of B[a]P on survival. Compared with the control HCC group, the survival curves of B[a]P-exposed, HCC-bearing mice declined significantly, suggesting that environmental B[a]P exposure contributes to the poor prognosis of HCC patients.

We observed that the NF-κB signaling pathway was involved in B[a]P-induced HCC metastasis, likely by activating the EMT cascade. NF-κB can transcriptionally activate the EMT inducer (snail) and subsequently regulate other effectors, such as E-cadherin. NF-κB has previously been implicated in EMT transition (Bonavida and Baritaki 2011). As a versatile transcription factor, NF-κB has been reported to induce MMP2 and MMP9 expression to promote HCC metastasis (Li et al. 2011). Whether this pathway is involved in B[a]P-induced HCC metastasis requires additional investigation.

For cancer progression, angiogenesis is essential to support the blood supply for tumor growth and metastasis (Weis and Cheresh 2011). We found that B[a]P increased HCC cell-induced recruitment of vascular endothelial cells and subsequent tube formation, possibly because the B[a]P-exposed HCC cells secreted more VEGF. Moreover, recruited vascular endothelial cells could in turn activate NF-κB signaling in HCC cells and facilitate metastasis and progression (Wang et al. 2013), which may serve as a positive feedback mechanism to enhance the long-term effects of B[a]P.

Our study has some limitations. It is generally reasonable that we used solvent-treated cells for the negative control groups, although cells with no treatment might have been better. Although B[a]P was metabolized to BPDE in HCC cells, to what extent BPDE mediates the effects of B[a]P on HCC progression remains unknown. Further, it is not known how B[a]P activates NF-κB signaling in HCC cells or whether other signaling pathways are involved in the cumulative effects of B[a]P. These possibilities need further investigation.

## Conclusions

A long-term exposure model based on human HCC cells was established and used to determine the adverse effects of B[a]P. In this model, B[a]P inhibited HCC cell adhesion and promoted migration and invasion. In mice, exposure of cells to B[a]P prior to injection enhanced HCC metastasis and decreased their survival. In addition, sustained B[a]P exposure enhanced the angiogenicity of HCC cells. The NF-κB pathway, which was involved in this process, might be the adverse outcome pathway. These findings suggest the cumulative toxicity of B[a]P on HCC cell angiogenesis and metastasis.

## Supplemental Material

(1.2 MB) PDF
