# Supplementary material for: Effects of Benzo[a]pyrene Exposure on Human Hepatocellular Carcinoma Cell Angiogenesis, Metastasis, and NF-κB Signaling
Source: Environ Health Perspect. 2014 Oct 17;123(3):246–54. doi: 10.1289/ehp.1408524 (PMC4348747; doi:10.1289/ehp.1408524)
Supplement: (1.2 MB) PDF [file ehp.1408524.s001.508.pdf]

Environmental Health Perspectives doi:10.1289/ehp.1408524

Supplemental Material

Effects of Benzo[*a*]pyrene Exposure on Human Hepatocellular Carcinoma Cell Angiogenesis, Metastasis, and NF-κB Signaling

Qian Ba, Junyang Li, Chao Huang, Hongling Qiu, Jingquan Li, Ruiai Chu, Wei Zhang, Dong Xie, Yongning Wu, and Hui Wang

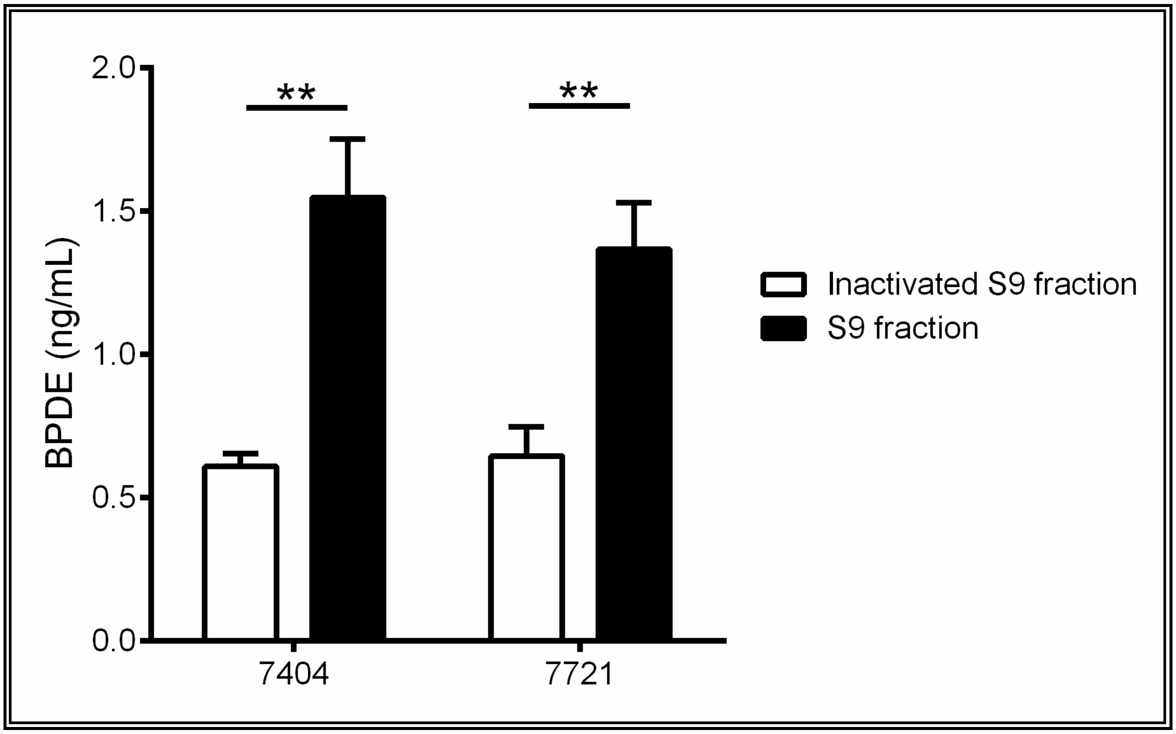

Figure S1. BEL-7404 and SMMC-7721 cells metabolized B(a)P. After B(a)P (10 μg/ml) was incubated in S9 reaction systems for 2 hr, the concentrations of BPDE in different groups were determined by ELISA. Data are presented as mean ± SD and analyzed by Student's *t*-test (n = 3/group).

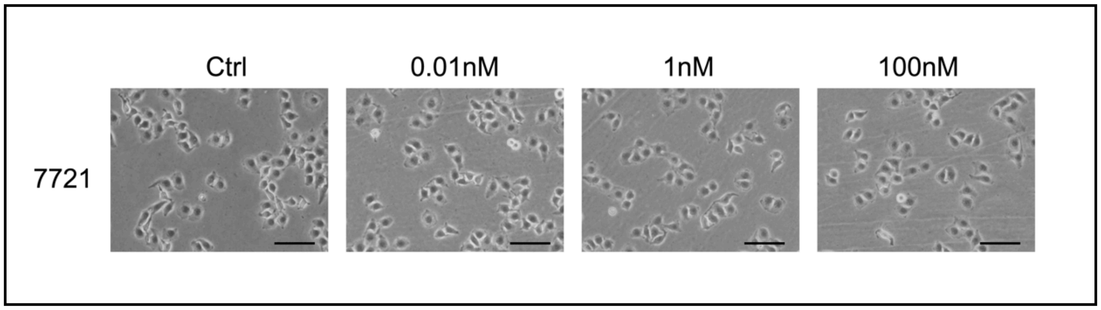

Figure S2. Long-term exposure to B(a)P did not alter HCC cell morphology. After exposed to different concentrations of B(a)P for a month, SMMC-7721 cells were photographed with a microscope. Scale bar, 100 μm.

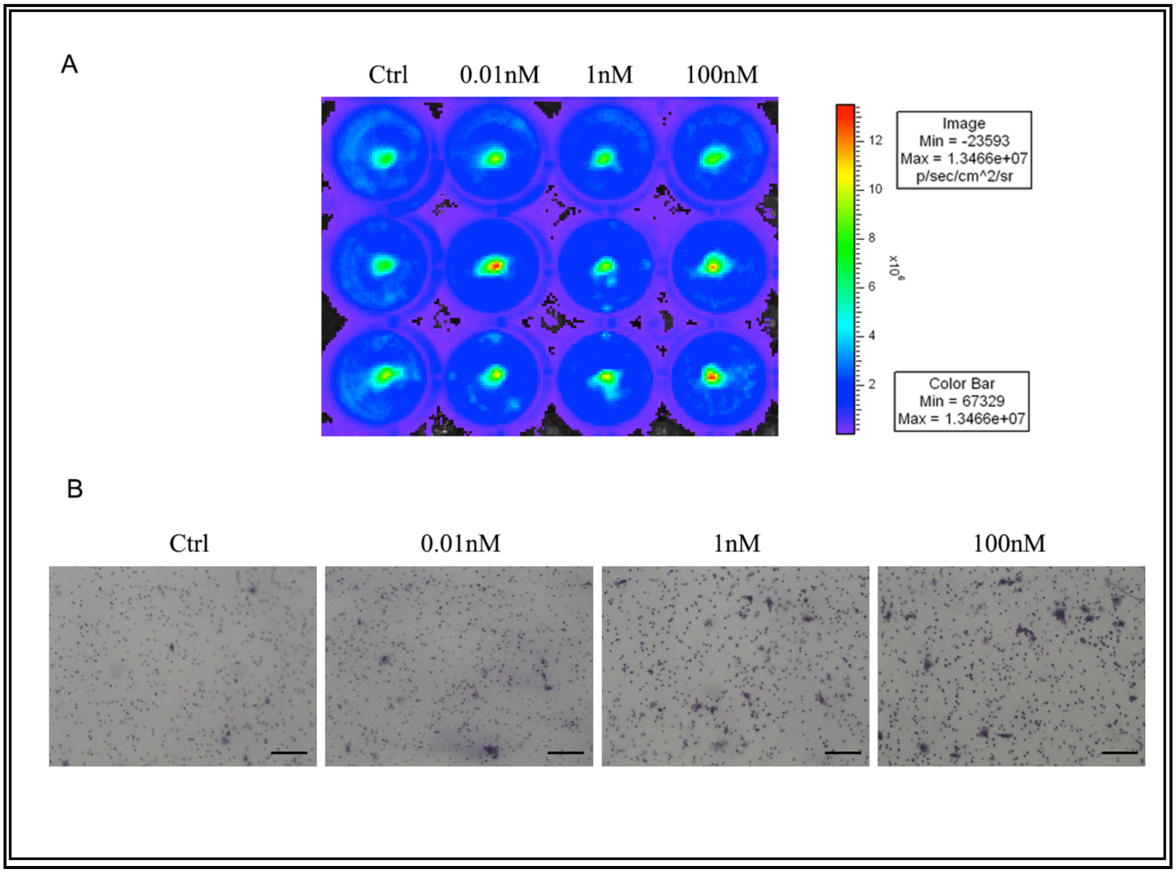

Figure S3. Lentivirus infection did not affect cell migration. (A) After virus infection, the luciferase activity of each group of B(a)P-exposed SMMC-7721 cells was determined by luciferin intensity. (B) After being labeled with luciferase, cell migration assays were performed; representative images were shown. Scale bar, 150 μm.
